# RAD51 is a poor prognostic marker and a potential therapeutic target for oral squamous cell carcinoma

**DOI:** 10.1186/s12935-023-03071-w

**Published:** 2023-10-05

**Authors:** Yu-Fen Tsai, Leong-Perng Chan, Yuk-Kwan Chen, Chang-Wei Su, Ching-Wei Hsu, Yen-Yun Wang, Shyng-Shiou F. Yuan

**Affiliations:** 1 Graduate Institute of Medicine, College of Medicine, Kaohsiung Medical University, Kaohsiung, 807 Taiwan; 2 Department of Hematology and Oncology, E-Da Cancer Hospital, I-Shou University, Kaohsiung, 824 Taiwan; 3 School of Chinese Medicine for Post Baccalaureate, College of Medicine, I-Shou University, Kaohsiung, 824 Taiwan; 4 Cohort Research Center, Kaohsiung Medical University, Kaohsiung, 807 Taiwan; 5 Faculty of Medicine, College of Medicine, Kaohsiung Medical University, Kaohsiung, 807 Taiwan; 6 Department of Otorhinolaryngology-Head and Neck Surgery, Kaohsiung Municipal Ta-Tung Hospital, Kaohsiung Medical University Hospital, Kaohsiung, 807 Taiwan; 7 School of Dentistry, College of Dental Medicine, Kaohsiung Medical University, Kaohsiung, 807 Taiwan; 8 Division of Oral Pathology & Maxillofacial Radiology, Kaohsiung Medical University Hospital, Kaohsiung, 807 Taiwan; 9 Drug Development and Value Creation Research Center, Kaohsiung Medical University, Kaohsiung, 807 Taiwan; 10 Department of Medical Research, Kaohsiung Medical University Hospital, Kaohsiung, 807 Taiwan; 11 Department of Biological Science and Technology, Institute of Molecular Medicine and Bioengineering, Center for Intelligent Drug Systems and Smart Bio-devices (IDS2B), National Yang Ming Chiao Tung University, 75 Bo-Ai Street, Hsinchu, 300 Taiwan; 12 Translational Research Center, Kaohsiung Medical University Hospital, Kaohsiung, 807 Taiwan; 13 Department of Obstetrics and Gynecology, Kaohsiung Medical University Hospital, Kaohsiung, 807 Taiwan

**Keywords:** Oral squamous cell carcinoma, RAD51, B02, Chemotherapy and radiotherapy resistance

## Abstract

**Objectives:**

RAD51 overexpression has been reported to serve as a marker of poor prognosis in several cancer types. This study aimed to survey the role of RAD51 in oral squamous cell carcinoma and whether RAD51 could be a potential therapeutic target.

**Materials and methods:**

RAD51 protein expression, assessed by immunohistochemical staining, was used to examine associations with survival and clinicopathological profiles of patients with oral squamous cell carcinoma. Lentiviral infection was used to knock down or overexpress RAD51. The influence of RAD51 on the biological profile of oral cancer cells was evaluated. Cell viability and apoptosis after treatment with chemotherapeutic agents and irradiation were analyzed. Co-treatment with chemotherapeutic agents and B02, a RAD51 inhibitor, was used to examine additional cytotoxic effects.

**Results:**

Oral squamous cell carcinoma patients with higher RAD51 expression exhibited worse survival, especially those treated with adjuvant chemotherapy and radiotherapy. RAD51 overexpression promotes resistance to chemotherapy and radiotherapy in oral cancer cells in vitro. Higher tumorsphere formation ability was observed in RAD51 overexpressing oral cancer cells. However, the expression of oral cancer stem cell markers did not change in immunoblotting analysis. Co-treatment with RAD51 inhibitor B02 and cisplatin, compared with cisplatin alone, significantly enhanced cytotoxicity in oral cancer cells.

**Conclusion:**

RAD51 is a poor prognostic marker for oral squamous cell carcinoma. High RAD51 protein expression associates with resistance to chemotherapy and radiotherapy. Addition of B02 significantly increased the cytotoxicity of cisplatin. These findings suggest that RAD51 protein may function as a treatment target for oral cancer.

**Trial registration:**

Number: KMUHIRB-E(I)-20190009 Kaohsiung Medical University Hospital, Kaohsiung, Taiwan, approved on 20190130, Retrospective registration.

**Supplementary Information:**

The online version contains supplementary material available at 10.1186/s12935-023-03071-w.

## Introduction

The incidence, mortality, and epidemiology of oral cancers vary significantly globally. The International Agency for Research on Cancer’s (IARC’s) Global Cancer Observatory (GLOBOCAN) reported 377,713 new cases and 177, 757 deaths from lip and oral cavity cancers worldwide in 2020 [1, 2], and its incidence is projected to increase around 50% from 2020 to 2040 [3]. In Unites States, according to Cancer Facts & Fig. 2023 from American Cancer Society, the five-year relative survival rates of oral cavity& pharynx cancer from 2012 to 2018 for local, regional, and distant state at diagnosis were 86%, 69%, and 40%, individually [4]. According to WHO report, South-East Asia Region has the highest incidence and mortality in oral cancer, with rates almost double the global average [5]. In Taiwan, oral cavity cancers are the third most common cancer and the fourth leading cause of cancer death in males, according to the 2019 Cancer Registry Annual Report from the Health Promotion Administration Ministry of Health and Welfare, Taiwan [6]. Oral squamous cell carcinoma (OSCC) is the most frequent malignancy in oral cancer. The treatment for OSCC is generally a multidisciplinary treatment including surgery, chemotherapy, and radiotherapy, and has a disappointing survival rate. The five-year overall survival rate in stage I, II, III, and IV oral cancers were 78.98%, 69.38%, 54.62%, and 36.17% individually, from a 10-year (2002–2011) Taiwan National Cancer Registry data without significant improvement [7].

DNA damage response (DDR) and repair pathways evolve a complex network of signal cascades to maintain genomic integrity, cell survival, and normal function. The DDR and repair pathways identify DNA damage, initiate cell cycle arrest, DNA repair, and apoptosis. There are several forms of DNA damage with DNA double-strand breaks (DSBs) the most fatal form. In eukaryotic cells, there are two major DSB repair mechanisms, homologous recombination (HR) and non-homologous end-joining (NHEJ) [8–11]. Defects in homologous recombination repair (HRR) contribute to genomic instability and lead to tumor development [12, 13].

RAD51, a DNA repair protein, plays a central role in HRR. During HRR, RAD51 is involved in the strand invasion and homologous pairing process. RAD51 forms nucleoprotein filaments and facilitates homology searching and strand invasion [14, 15]. RAD51 is regulated by proteins include BRCA2, PALB2, and the RAD51 paralogs. BRCA2 and PALB2 binds to RAD51 and these interactions enhance strand invasion activity [16–18]. Additionally, RAD51 expression is regulated by several transcription factors, including Cyclin-dependent kinases (CDK), members of the E2F Transcription Factor Family, P53, and several RAD51 cofactors including RAD51C, RAD51B, RAD51D, XRCC1 and XRCC2 [19–22]. Previous studies have revealed that CDK4/6 were overexpressed in OSCC tissues and cancer cells and were linked to oral carcinogenesis [23]. Moreover, targeting CDK4/6 induces senescence and inhibits DNA damage repair including both HR and NHEJ pathways [24].

RAD51 is overexpressed in many cancer cell lines [25, 26] and primary tumors, including breast [27], pancreatic [28], prostate [29], non-small cell lung [30], and esophageal cancers [31, 32]. Several studies have shown that RAD51 expression is associated with resistance to radiotherapy and chemotherapy in osteosarcoma and lung cancer cells [33–36]. In addition, elevated RAD51 protein expression has been found to be associated with poor survival in a variety of tumors, including ovarian cancers [37], lung cancers [38, 39], pancreatic cancers [40], esophageal cancers [32], neuroblastoma [41], and breast cancers [42].

The information regarding the role of RAD51 in OSCC was limited. In a previous study, RAD51 expression was found higher in tumor cells than in normal tissues and elevated RAD51 expression was associated with poor differentiation, lymphatic metastases, and higher relapse rates in clinical study [43]. However, the mechanisms of higher relapse rate in OSCC patients with elevated RAD51 expression and biology profiles of RAD51 protein in OSCC cells are still unclear and warrant further investigation. Therefore, we designed a study to examine RAD51 protein expression in OSCC tissues and investigate its association with clinicopathological parameters and survivals. Also, RAD51 was knockdown and overexpressed to survey its impact on biology profiles and survivals in OSCC cells after chemotherapy or irradiation. Furthermore, we explored whether RAD51 could be a potential novel target to overcome the high relapse rate and poor outcome in OSCC patients.

## Materials and methods

### Clinical samples

A total of 105 patients with OSCC diagnosed at Kaohsiung Medical University Hospital (KMUH) were included in the study. Newly diagnosed OSCC patients with age more than 20 years old who received first line treatment with surgery were enrolled in this study. Patients who had been exposed to chemotherapy or radiotherapy or with distant metastasis were excluded from the study. Clinical samples were collected at the initial surgery without preoperative chemotherapy or radiotherapy and clinical data were collected from patients’ medical records. The staging was based on the 8th edition American Joint Cancer Committee on Cancer (AJCC) Staging System [44]. The Institutional Review Board of Kaohsiung Medical University Hospital, Kaohsiung, Taiwan, in 2019 (KMUHIRB-E(I)-20190009) was approved for this study.

### Immunohistochemistry (IHC) and immunohistochemical evaluation

Formalin-fixed, paraffin-embedded (FFPE) tissues were used to determine the immunohistochemical expression of RAD51. Automatic IHC staining device was used for immunohistochemical analyses, according to the manufacturer’s protocol (Bond-Max Automated Immunostainer; Vision Biosystems, Melbourne, Australia). The primary antibody used was a rabbit anti-human RAD51 polyclonal antibody (GTX100469, GeneTex). A negative control was obtained by omitting the primary antibody. Only the nuclear staining of RAD51 was counted. The proportion of positively stained tumor cells, the positive stained-cell index (PCI), was determined by assessing tumor sections, with each sample assigned to one of the following scores: 0 (0–10% positivity), 1 (10–25% positivity), 2 (25–50% positivity), 3 (50–75% positivity), or 4 (75–100% positivity) [43]. For further statistical analysis, PCI ≦ 10% was categorized as low expression and PCI > 10% as high expression [32, 39]. The expression of RAD51 was viewed and evaluated by two independent researchers, who were blinded to the clinicopathological data of OSCC patients. In rare cases, the discordant scores were re-evaluated and discussed by the two researchers to obtain a consensual conclusion.

### Cell culture and reagents

SAS, Ca9-22, CAL 27, HSC-3 and OECM1 are human oral squamous cancer cell lines from the Bioresource Collection and Research Center, Taiwan (www.bcrc.firdi.org.tw). The cells were cultured in media from Thermo Fisher Scientific (Waltham, MA, USA) with 10% (v/v) fetal bovine serum (FBS) (Biological Industries, Haemek, Israel) and antibiotics (100 µg/mL streptomycin, 100 units/mL penicillin, and 2.5 µg/mL amphotericin B) (Biological Industries, Haemek, Israel). All cells were grown at 37 °C in a 5% CO2 incubator. Cisplatin (Sigma-Aldrich), bleomycin (Sigma-Aldrich), and mitomycin (Sigma-Aldrich) were dissolved in normal saline, and B02 (Sigma-Aldrich) was dissolved in DMSO and diluted in cell culture medium for cell treatment.

### Virus infection for RAD51 knockdown/overexpression

RAD51 was silenced in OSCC cells with a pLKO.1_puro lentiviral vector expressing shRNA oligonucleotides targeting the sequence of human RAD51 (shRNA1, 5’- CGCCCTTTACAGAACAGACTA − 3’, National RNAi Core Facility, Academia Sinica, Taiwan). Another pLKO.1_puro lentiviral vector expressing shRNA targeting firefly luciferase unrelated to the human genome sequence was used as a negative control (National RNAi Core Facility, Academia Sinica, Taiwan).

The pReceiver Lv105 lentiviral vector expressing the human RAD51 gene from GeneCopoeia (Rockville, MD, USA) was used to overexpress RAD51 in OSCC cells, and an empty pReceiver Lv105 lentiviral vector (GeneCopoeia, Rockville, MD, USA) was used for the negative control. Viral solution containing 8 µg/mL polybrene and 2 µg/mL puromycin was added in culture media for lentiviral infection. The surviving cells were maintained continuously with 2 µg/mL puromycin.

### XTT cell viability assay

XTT (tetrazolium salt 2,3-bis[2-methyloxy-4-nitro-5-sulfophenyl]-2 H-tetrazolium-5- carboxanilide) assay was evaluated for the effect of RAD51 on OSCC cell proliferation. SAS cells with RAD51 knockdown and OECM1 cells with RAD51 overexpression were seeded (5,000 cells/well) in 96-well culture plates. The medium was removed at 48 and 72 h after treatment with chemotherapeutic agents, B02, irradiation, and then 100µL of XTT solution (Sigma-Aldrich, St. Louis, MO, USA) was added and incubated at 37 °C for 2 h. The result was obtained by measuring the optical density (OD) at 475 nm and subtracting the nonspecific background at 660 nm (OD475 nm - OD660 nm).

### In vitro migration assays

Cell migration assays were performed using Transwell (Corning Costar Corp., Cambridge, MA, USA) membrane filter inserts in 24-well tissue culture plates (6.5 mm diameter, 8 μm pore size). Oral cancer cells with RAD51 knockdown or overexpression were suspended in serum-free medium and seeded in the upper chamber of the transwell filters. Serum-containing medium was added to the lower chamber and incubated at 37 °C for 24 h. Then, the cells were fixed with 4% formaldehyde and stained with crystal violet. Non-migrating cells were removed. The number of migrated cells was determined using the ImageJ software.

### Immunoblotting analysis

Immunoblotting analysis was performed as described in a previous article [45]. Western blot images were acquired using a chemiluminescence reagent (WBKLS0500, Merck Millipore) and then quantified using the ChemiDoc XRS + imaging system (BIO-RAD). The antibodies used were as follows: α-tubulin (GTX112141, GeneTex), RAD51 (GTX100469, GeneTex), γ-H2AX (GTX61796, GeneTex), CD44 (5640, Cell Signaling Technology), Oct-4 (GTX101497, GeneTex), SOX2 (GTX101507, GeneTex), Nanog (GTX100863, GeneTex), and CD133 (GTX100567, Genetex). HRP-conjugated goat anti-rabbit and anti-mouse antibodies were obtained from GeneTex (Irvine, CA).

### Colony forming assay

OECM1 cells overexpressing RAD51 were seeded in 6-well plates (5,000 cells/well), exposed to 2, 5, or 10 Grays (Gy) irradiation, and incubated at 37 °C for 7 days. The cells were washed twice with phosphate-buffered saline (PBS) and stained with 0.1% crystal violet for 15 min. Clusters containing more than 50 cells were counted as colonies. The experiments were repeated 3 times.

### Annexin V staining

The FITC Annexin V Apoptosis Detection Kit I (BD Biosciences, San Jose, CA) was used to detect apoptotic cells after irradiation or cisplatin treatment. OECM1 cells overexpressing RAD51 were seeded on 6 cm plates. At 24, 48, and 72 h after irradiation or cisplatin treatment, 1 × 10^6^cells were collected for Annexin V staining. The protocol was performed as described in a prior article and analyzed in three independent experiments [46].

### Tumorsphere formation assay

OECM1 cells were isolated following pretreatment and resuspended in DMEM/F12 (Gibco D8437). Additional recombinant human fibroblast growth factor basic (20 ng/mL) (Sigma F0291), recombinant human epidermal growth factor (20 ng/mL) (Sigma E9644), 1xB27 (Gibco 17-504-044), and insulin (10 µg/mL; Sigma I3536) were added. Cells (4 × 10^3^) were seeded per well, plated onto an ultra-low-attachment 24-well plate (Corning), and cultured for seven days. Tumorspheres more than 50 μm in diameter were counted as positive.

### Statistical analysis

For descriptive statistics, the clinicopathological variables of patients with OSCC were presented as percentages, frequencies, and means ± standard deviation (SD). Chi-square or Fisher’s exact test were used for categorical variables; Student’s t-test or nonparametric tests were used for statistically significant differences in continuous variables between RAD51 protein high and low expression groups. The Cox proportional hazard model was used to evaluate associations between clinicopathological factors and survival in univariate and multivariable analyses. In the multivariable analysis, variables with P-values < 0.05 in the univariate analysis were included as covariates. The Kaplan-Meier method was used to estimate overall survival (OS) and progression-free survival (PFS), and survival curves were compared using the log-rank test. Two-sided Student’s t-tests were used for in vitro studies. Statistical significance was a two-tailed P-value < 0.05.

## Results

### Baseline characteristics of OSCC patients and RAD51 expression in OSCC tissues

**Fig. 1:**
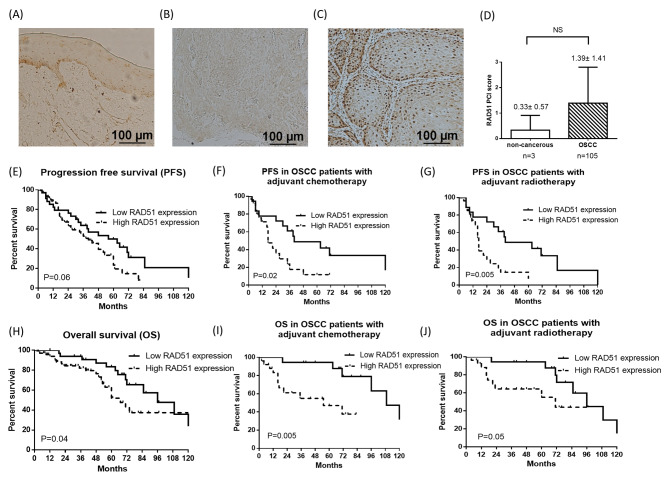
RAD51 expression is higher in oral cancer tissues and associated with poor patient prognosis. (**A**) Immunohistochemical stain of RAD51 (100x). Representative photo for RAD51 expression in oral non-cancerous tissues. (**B**) Representative photo for low RAD51 expression in oral cancer tissues. (**C**) Representative photo for high RAD51 expression in oral cancer tissues. (**D**) Quantitative result for RAD51 expression in oral non-cancerous and cancer tissues. (**E**) Progression free survival (PFS) in OSCC patients stratified by RAD51 expression and high RAD51 expression had a trend of shorter PFS. (**F**) PFS in oral squamous cell carcinoma (OSCC) patients receiving adjuvant chemotherapy stratified by RAD51 expression and high RAD51 expression had a significant shorter PFS. (**G**) PFS in OSCC patients receiving adjuvant radiotherapy stratified by RAD51 expression and high RAD51 expression had a significant shorter PFS. (**H**) Overall survival (OS) in OSCC patients stratified by RAD51 expression and high RAD51 expression had a worse OS. (**I**) OS in OSCC patients receiving adjuvant chemotherapy stratified by RAD51 expression and high RAD51 expression had a significant worse OS. (**J**) OS in OSCC patients receiving adjuvant radiotherapy stratified by RAD51 expression and high RAD51 expression had a borderline worse OS. NS, not significant, PCI, positive stained-cell index

A total of 105 cases were enrolled in the IHC analysis of RAD51 protein expression in OSCC tissues, and the typical photos for high and low nuclear RAD51 protein expression in OSCC tissues and non-cancerous oral epithelial tissue are shown in Fig. 1A-C. The mean expression score of RAD51 in OSCC was higher than oral non-cancerous tissue (1.39 versus 0.33) but the difference was not statistically significant (Fig. 1D). The clinicopathological characteristics of the 105 patients are presented in Table S.1. Most of patients were male (92.4%). The median age was 53 years. Approximately three-quarters of the patients had habits of alcohol consumption, betel nut chewing, and cigarette smoking. 60% of the patients were in the early stages (stages I and II), and 78.1% of the patients were without lymph node metastasis. More than half of the patients did not receive adjuvant chemotherapy or radiotherapy postoperatively. High RAD51 expression was observed in 67.6% (34 out of 105) of the patients.

### The correlation of RAD51 expression to clinicopathological parameters in OSCC patients

The correlation between RAD51 protein expression and the clinicopathological parameters is shown in Table S.2. We found no significant correlation between RAD51 expression and patient age, sex, tumor stage (T stage), lymph node stage (N stage), clinical stage, tumor grade, betel nut chewing, or cigarette smoking. However, a positive correlation was observed between alcohol consumption and RAD51 expression. Patients whose cancer tissues showed high RAD51 expression, in comparison to low RAD51 expression, had a higher percentage of alcohol consumption (84.1% vs. 63.6%, P = 0.02).

### RAD51 expression is negatively correlated with progression free survival and overall survival in OSCC patients, especially in those received adjuvant chemotherapy and radiotherapy

The expression of RAD51 in oral cancer tissues, determined by immunohistochemistry, was negatively correlated with PFS and OS, and the Kaplan–Meier survival curves showed that the low RAD51 expression group had better PFS and OS than the high RAD51 expression group with P values of 0.06 and 0.04, respectively (Fig. 1E H). In subgroup analyses, PFS was markedly shorter in the high RAD51 expression group who received adjuvant chemotherapy and radiotherapy, with P values of 0.02 and 0.005, respectively (Fig. 1F and G). In addition, OS was worse in the high RAD51 expression group that received adjuvant chemotherapy and radiotherapy, with P values of 0.005 and 0.05, respectively (Fig. 1I J). RAD51 protein expression did not have prognostic value for PFS and OS in patients with OSCC who did not receive adjuvant chemotherapy or radiotherapy.

### RAD51 was an independent risk factor for PFS and OS in OSCC patients receiving adjuvant chemotherapy and radiotherapy

We performed Cox regression analyses of PFS in OSCC patients who received adjuvant chemotherapy and found that RAD51 expression was the only independent risk factor for PFS, with a hazard ratio (HR) of 2.36 and a P value of 0.035. (Table 1 A). OS in patients with OSCC receiving adjuvant chemotherapy was also analyzed using univariate and multivariable Cox regression analyses. Both stage and RAD51 expression were risk factors for OS. Advanced stage (HR = 3.39, P = 0.029) and high RAD51 expression (HR = 5.27, P = 0.013) were associated with poor OS (Table 1B). In addition, PFS in OSCC patients receiving adjuvant radiotherapy also showed that RAD51 expression was the only independent risk factor with a HR of 3.00 and a P value of 0.008 (Table 2 A). However, the high RAD51 expression group displayed only a marginally worse OS in OSCC patients receiving adjuvant radiotherapy (HR = 2.67, P = 0.069) (Table 2B).

**Table 1:** RAD51 was an independent risk factor for PFS and OS in OSCC patients receiving chemotherapy

(A)	Univariate analysis of PFS		(B)	Univariate analysis of OS		Multivariable analysis of OS	
Variables	HR (95%CI)	P-value	Variables	HR (95%CI)	P-value	HR (95%CI)	P-value
**Sex**			**Gender**				
Male	1.00		Male	1.00			
Female	0.04 (0.00-13.52)	0.282	Female	0.05 (0.0-852.71)	0.538		
**Age**	1.01 (0.98–1.05)	0.478	**Age**	1.02 (0.97–1.08)	0.398		
**Alcohol consumption**	0.93 (0.42–2.07)	0.859	**Alcohol consumption**	1.66 (0.46–5.99)	0.437		
**Betel nut chewing**	1.53 (0.55–4.07)	0.395	**Betel nut chewing**	1.53 (0.34–6.87)	0.581		
**Cigarette smoking**	0.83 (0.37–1.90)	0.663	**Cigarette smoking**	1.35 (0.37–4.84)	0.650		
**Stage**		0.702	**Stage**		0.029*		0.029*
I, II	1.00		I, II	1.00		1.00	
III, IV	0.87 (0.41–1.82)		III, IV	3.34 (1.13–9.82)		3.39 (1.13–10.13)	
**RAD51 expression**		0.035*	**RAD51 expression**		0.013*		0.013*
Low expression	1.00		Low expression	1.00		1.00	
High expression	2.36 (1.06–5.23)		High expression	5.18 (1.42–18.87)		5.27 (1.43–19.47)	

(A). Univariate Cox regression analysis of Progression free survival (PFS) in OSCC patients receiving adjuvant chemotherapy and RAD51 was the only independent risk factor for PFS. (B). Univariate and multivariate Cox regression analyses of overall survival (OS) in OSCC patients receiving adjuvant chemotherapy. Stage and RAD51 expression were the two poor prognostic factors for OS

*PFS, progression-free survival; OS, overall survival; OSCC, oral squamous cell carcinoma; HR, hazard ratio; CI, confidence interval

**Table 2:** **RAD51 protein was a poor prognostic factor for PFS in OSCC patients receiving adjuvant radiotherapy**

(A)	Univariate analysis of PFS		(B)	Univariate analysis of OS	
Variables	HR (95%CI)	P-value	Variables	HR (95%CI)	P-value
**Sex**			**Sex**		
Male	1.00		Male	1.00	
Female	0.73 (0.22–2.46)	0.610	Female	1.19 (0.27–5.27)	0.823
**Age**	1.02 (0.99–1.05)	0.303	**Age**	1.03 (0.98–1.08)	0.262
**Alcohol consumption**	0.74 (0.35–1.55)	0.423	**Alcohol consumption**	2.27 (0.64–8.07)	0.204
**Betel nut chewing**	1.47 (0.59–3.61)	0.406	**Betel nut chewing**	1.60 (0.36–7.18)	0.538
**Cigarette smoking**	0.95 (0.46–1.96)	0.888	**Cigarette smoking**	0.82 (0.29–2.33)	0.707
**Stage**		0.718	**Stage**		0.492
I, II	1.00		I, II	1.00	
III, IV	0.88 (0.43–1.78)		III, IV	1.39 (0.54–3.58)	
**RAD51 expression**		0.008*	**RAD51 expression**		0.069
Low expression	1.00		Low expression	1.00	
High expression	3.00 (1.33–6.78)		High expression	2.67 (0.93–7.68)	

(A). Univariate Cox regression analysis of progression free survival in OSCC patients with adjuvant radiotherapy. (B). Univariate Cox regression analysis of overall survival in OSCC patients with adjuvant radiotherapy

*PFS, progression-free survival; OS, overall survival; OSCC, oral squamous cell carcinoma; HR, hazard ratio; CI, confidence interval

### RAD51 protein had no effect on oral cancer cell proliferation and migration

RAD51 expression in different oral cancer cell lines (Ca9-22, CAL27, SAS, HSC-3, and OECM1) was determined by western blotting. SAS showed the highest RAD51 expression, whereas OECM1 showed the lowest RAD51 expression. Therefore, we chose SAS to knockdown RAD51 expression and OECM1 to overexpress RAD51 by lentivirus transduction to analyze the function of RAD51 in oral cancer cells. The RAD51 expression levels are shown in Fig. S.1.

We also investigated the influence of RAD51 expression on the biological behavior of oral cancer cells. No significant difference in cell viability or migration was observed in RAD51 overexpression or knockdown cells compared to control cells (Fig. S.2 A-D).

### RAD51 expression was associated with radio-resistance and chemo-resistance

**Fig. 2:**
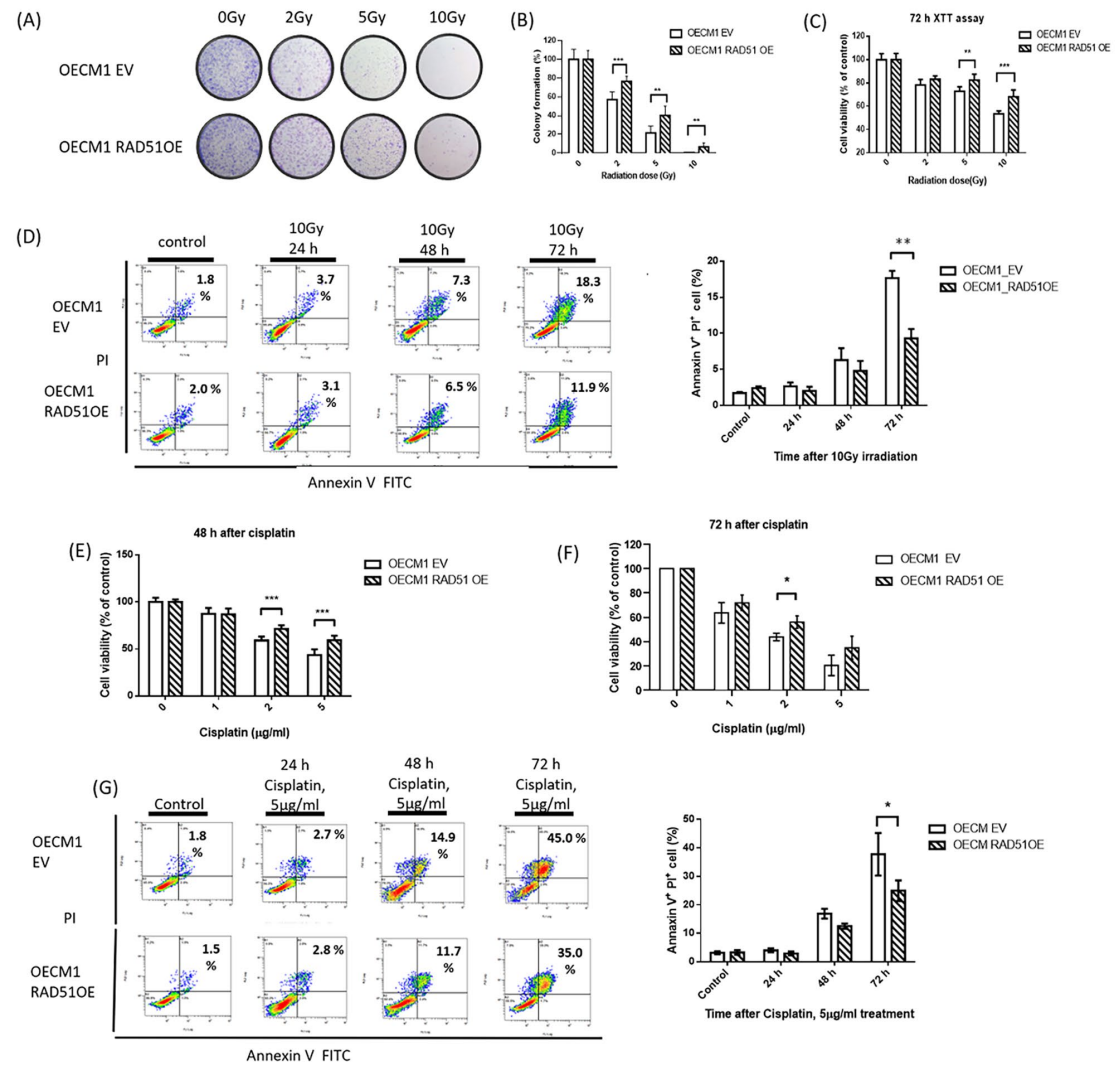
RAD51 overexpressing oral cancer cells survived better with less apoptotic cells after irradiation and cisplatin. (**A**) Colony formation assay in human oral cavity squamous cell carcinoma cell line, OECM1 EV (control) and OECM1 RAD51 OE (overexpression) cells after different doses of irradiation, and OECM1 RAD51 OE cells had more colonies after irradiation, (**B**) The quantification of colony formation showed that OECM1 RAD51 OE cells had better survival after different doses of irradiation than OECM1 EV cells, (**C**) XTT assay in OECM1 EV and OECM1 RAD51 OE cells at 72 h after irradiation showed that OECM1 RAD51 OE cells had higher cell viability after irradiation, (**D**) OECM1 EV and OECM1 RAD51 OE cells were stained with a combination of annexin V and PI and analyzed by FACS. Cells positive for annexin V and PI staining were counted as apoptotic cells. The bar chart describes the percentage distribution of apoptotic cells. OECM1 RAD51 OE cells have less apoptotic cells at 72 h after 10 Gy irradiation than OECM1 EV cells, (**E**) XTT assay in OECM1 EV and OECM1 RAD51 OE cells at 48 h after treatment with difference doses of cisplatin and RAD51 OE cells survived better after cisplatin treatment, (**F**) XTT assay in OECM1 EV and OECM1 RAD51 OE cells at 72 h after cisplatin treatment and RAD51 OE cells survived better after cisplatin treatment, (**G**) OECM1 EV and OECM1 RAD51 OE cells were stained with a combination of annexin V and PI and analyzed by FACS. OECM1 RAD51 OE cells have less apoptotic cells at 72 h after treatment with 5 µg/ml cisplatin Gy, gray; *, P < 0.05; **, P < 0.01; ***, P < 0.001

To study the effect of RAD51 expression on radio- and chemo-resistance, colony formation assays were performed on OECM1 control cells (OECM1 EV) and OECM1 RAD51 overexpression cells (OECM1 RAD51 OE) after irradiation. The results showed that OECM1 RAD51 OE cells had greater colony formation after irradiation at different doses (Fig. 2A and B). XTT assay at 72 h after irradiation also showed that OECM1 RAD51 OE cells survived better than control cells, especially after 5 and 10 Gy irradiation (Fig. 2C). The annexin V/PI assay revealed that OECM1 RAD51 OE cells, compared to control cells, had less apoptotic cells (11.9% versus 18.3%) at 72 h after 10 Gy irradiation (Fig. 2D). γ-H2AX expression in OECM1 RAD51 OE cells, compared to control cells, was decreased, especially 24 h after 10 Gy irradiation (Fig. S.3).

For chemotherapy treatment, OECM1 RAD51 OE cells showed similar results as irradiation treatment. OECM1 RAD51 OE cells showed better survival after cisplatin, mitomycin, and bleomycin treatment (Fig. 2E F, and Fig. S.4 C-F). Annexin V/PI assay results also showed that OECM1 RAD51 OE cells, compared to control cells, had less apoptotic cells (35.0% versus 45.0%) at 72 h after 5 µg/ml cisplatin treatment (Fig. 2G). SAS RAD51 knockdown cells (SAS shRAD51) were more sensitive to mitomycin treatment than control cells (SAS shluc cells) (Fig. S.4 A and 4B). Another oral cancer cell line CAL27 also revealed similar result that RAD51 OE cells survived better after cisplatin and mitomycin treatment (Fig.S.5 A-D).

### RAD51 inhibitor, B02, enhanced the chemotoxicity both in OECM1 control and RAD51 overexpression cells

**Fig. 3:**
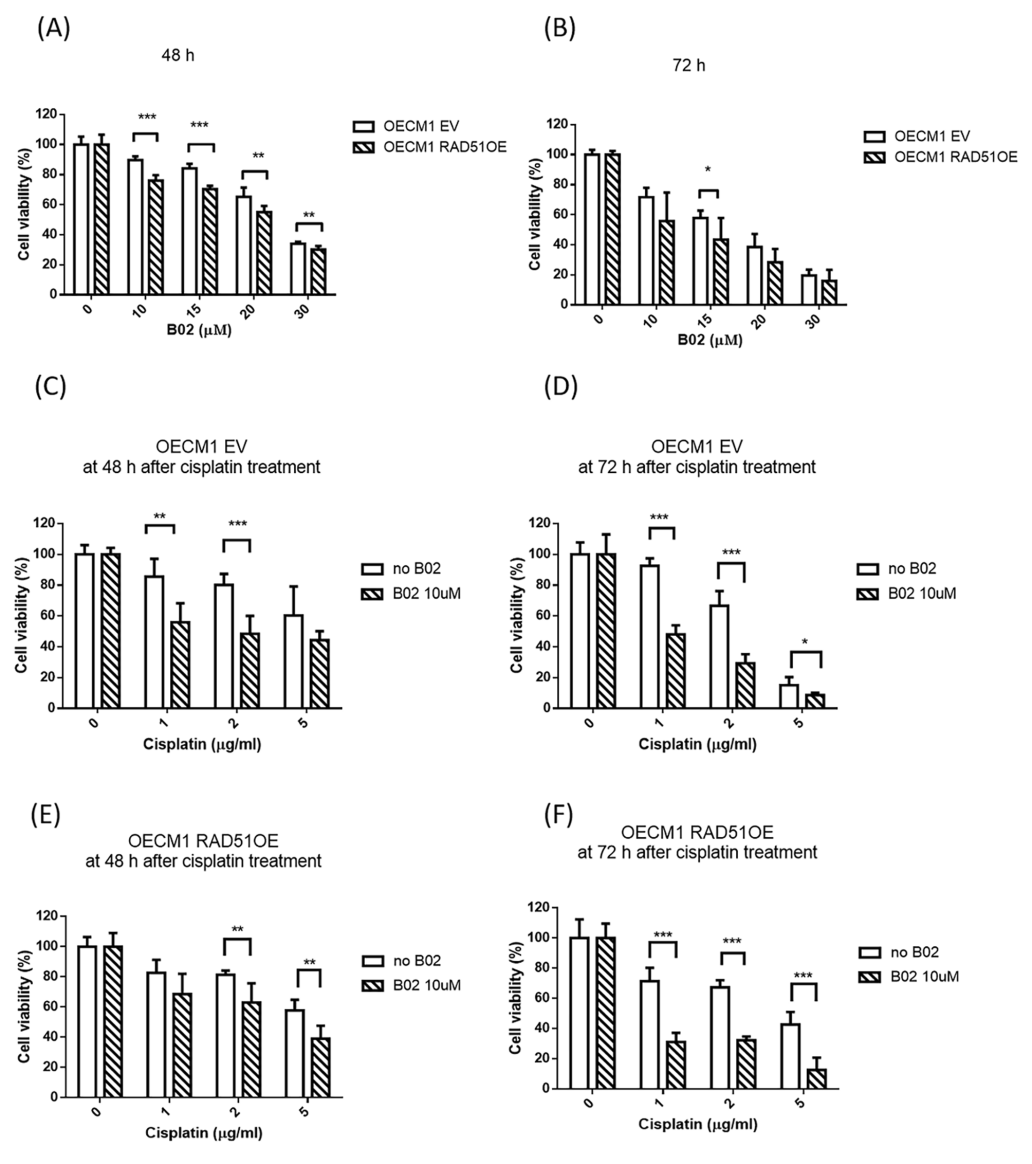
B02 significantly enhanced cytotoxicity of cisplatin in both OECM1 EV cells and RAD51 OE cells. (**A**) XTT assay in OECM1 EV and OECM1 RAD51 OE cells at 48 h after B02 treatment. B02 decreased cell viability both in control and RAD51 OE cells, (**B**) XTT assay in OECM1 EV and OECM1 RAD51 OE cells at 72 h after B02 treatment, (**C**) XTT assay in OECM1 EV cells at 48 h after cisplatin treatment with/without B02 and B02 enhanced cytotoxicity of cisplatin, (**D**) XTT assay in OECM1 EV cells at 72 h after cisplatin treatment with/without B02 and B02 enhanced cytotoxicity of cisplatin, (**E**) XTT assay in OECM1 RAD51 OE cells at 48 h after cisplatin treatment with/without B02 and B02 enhanced cytotoxicity of cisplatin, (**F**) XTT assay in OECM1 RAD51 OE cells at 72 h after cisplatin treatment with/without B02 and B02 enhanced cytotoxicity of cisplatin OECM1 EV, control; OECM1 RAD51 OE, overexpression; *, P < 0.05; **, P < 0.01; ***, P < 0.001

To study the cytotoxicity of RAD51 inhibition in oral cancer cells, RAD51 inhibitor B02 was added to OECM1 EV and OECM1 RAD51OE cells and showed dose-dependent cytotoxicity in both control and RAD51 overexpression cells. Moreover, higher cytotoxicity of B02 was observed in RAD51 overexpression cells than in control cells (Fig. 3A and B). To further study the effect of RAD51 inhibition on cytotoxicity caused by cisplatin, we designed two experimental groups. One group was treated with B02 and cisplatin added to OECM1 EV and OECM1 RAD51 OE cells, and the other group was treated with cisplatin alone. The XTT assay at 48 and 72 h showed that RAD51 inhibition significantly increased the cytotoxicity of cisplatin in both control and RAD51 overexpression cells. (Fig. 3C-F).

### Elevated RAD51 expression enhanced tumorsphere formation in oral cancer cells

**Fig. 4:**
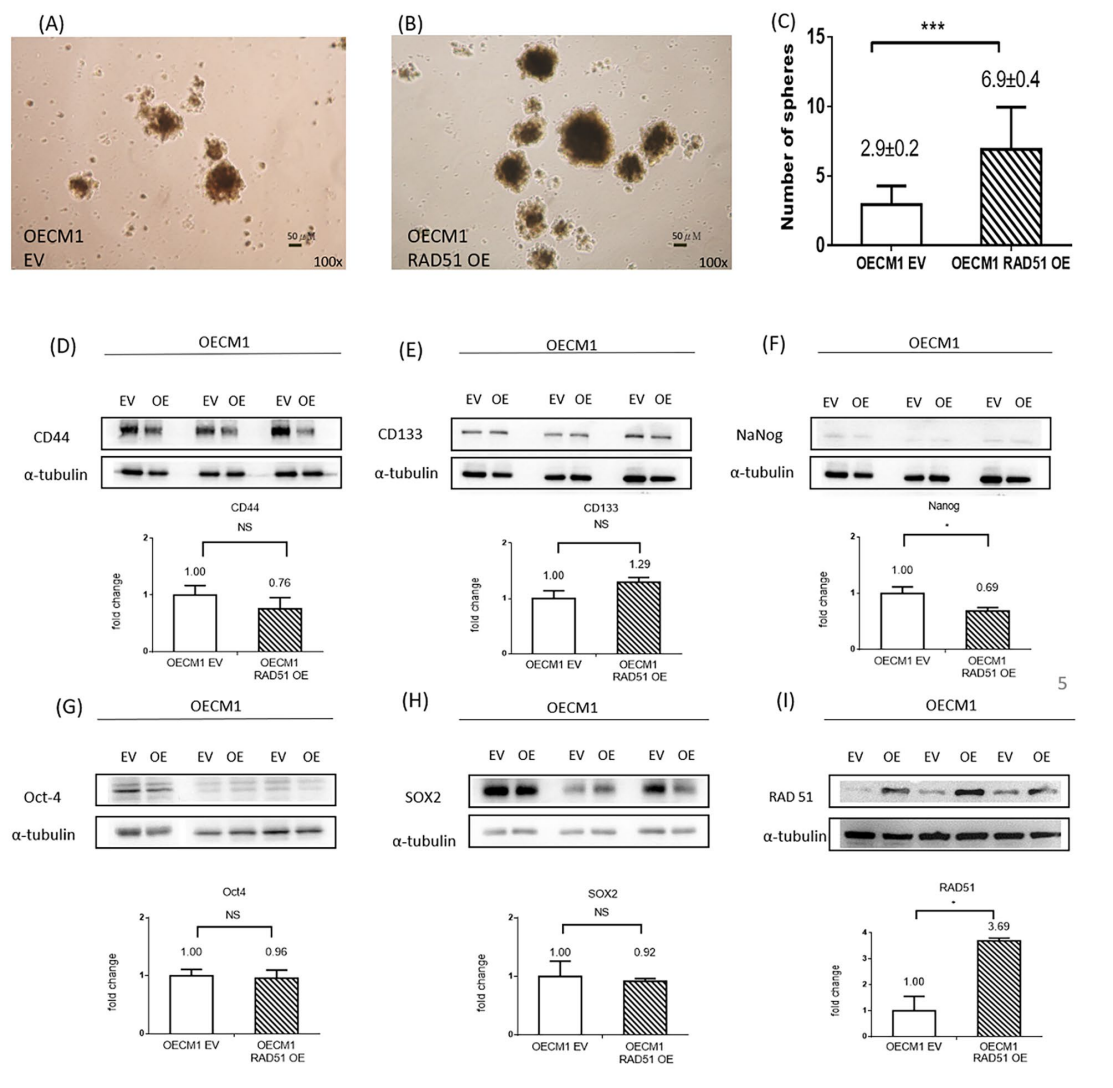
More tumorspheres were formed after RAD51 overexpression, however, no difference in expressions of stemness marker. Tumorsphere formation in (**A**) OECM1 EV (control) and (**B**) OECM1 RAD51 OE (overexpression) cells; (**C**) The bar chart describes the number of spheres formed in OECM1 EV and OECM1 RAD51 OE cells. More tumorspheres were formed in OECM1 RAD51 OE cells, (**D**) CD44 expression in OECM1 EV and OECM1 RAD51 OE cells, (**E**) CD133 expression in OECM1 EV and OECM1 RAD51 OE cells, (**F**) Nanog expression in OECM1 EV and OECM1 RAD51 OE cells, (**G**) Oct-4 expression in OECM1EV and OECM1 RAD51 OE cells, (**H**) SOX2 expression in OECM1 EV and OECM1 RAD51 OE cells, (**I**) RAD51 expression in OECM1 EV and OECM1 RAD51 OE cells NS, not significant; *, P < 0.05

To study the effect of RAD51 expression on cancer stemness, a tumorsphere formation assay was performed on OECM1 EV and OECM1 RAD51 OE cells. More tumorsphere formation was observed in OECM1 RAD51 OE cells than OECM1 EV cells (6.9 ± 0.4 versus 2.9 ± 0.2) (Fig. 4A-C). However, the expression of tumor stemness markers, including CD44, CD133, Nanog, Oct4, and SOX2, was similar between OECM1 EV and OECM1 RAD51 OE cells (Fig. 4D-I).

## Discussion

We found that RAD51 protein was a poor prognostic factor for OS and PFS in patients with OSCC who received adjuvant chemotherapy and radiotherapy. High RAD51 protein expression in oral cancer tissues had a poor impact on the OS of OSCC patients receiving adjuvant chemotherapy and a significantly negative influence on the PFS of OSCC patients receiving adjuvant radiotherapy. The negative influence of RAD51 expression on PFS and OS was not observed in patients with OSCC who did not receive adjuvant chemotherapy or radiotherapy. These results suggest that RAD51 protein expression is associated with resistance to chemotherapy and radiotherapy in patients with oral cancer, as confirmed by our in vitro studies. Oral cancer cells with higher RAD51 protein expression survived better after treatment with different chemotherapeutic agents, including cisplatin, mitomycin, and bleomycin, and after irradiation. Our findings agreed with prior studies that RAD51 levels were well correlated with resistance to etoposide in small cell lung cancer (SCLC) cells. Introduction of the exogenous RAD51 gene into etoposide-sensitive SCLC cells, which have low levels of RAD51, confers resistance to etoposide. Conversely, introduction of the antisense RAD51 gene into etoposide-resistant SCLC cells, which have a high level of RAD51, confers sensitivity to etoposide [34]. In addition, knockdown of RAD51 expression reversed drug resistance to gemcitabine in human non-SCLC cells and induced radio- and chemosensitivity in osteosarcoma cells [35].

It was reported that RAD51 expression increases after irradiation on non-small cell lung cancer cell lines H820 and A549 and on esophageal squamous cell carcinoma cell lines KYSE30 and KYS450 [47, 48]. Therefore, we excluded patients who had been exposed to chemotherapy or radiotherapy in our study to minimize the influence of neoadjuvant treatment on RAD51 expression levels to define more accurately the original RAD51 expression in tumor samples and investigate the associations between RAD51 expression and patient profiles including clinicopathologic parameters and clinical outcomes.

Several studies have also demonstrated an association between RAD51 protein expression, and aggressive proliferative and metastatic potential of malignancy. In a study of 70 neuroblastoma patients, RAD51 expression was significantly increased in stage 4 tumors compared to stage 1 and stage 2 tumors and was higher in bone marrow metastasis [41]. Another study found that breast cancer with lymph node metastases was associated with high RAD51 expression [42]. Inconsistent with these studies, our data showed no significant correlation between RAD51 expression and tumor stage (T stage), lymph node metastasis, clinical stage, and tumor differentiation. Our results might explain why RAD51 protein was a poor prognostic factor for OS and PFS only in patients with OSCC who received adjuvant chemotherapy and radiotherapy, but not in the overall patient population. These findings suggest that RAD51 expression is specifically related to treatment resistance rather than aggressive behavior in patients with OSCC.

We found a correlation between RAD51 expression and alcohol consumption. High RAD51 protein expression had higher percentages of alcohol consumption than low RAD51 protein expression (84.1% vs. 63.6%). Prior research has demonstrated that intake of alcohol seemed to result in DNA double-strand breaks and triggered a DNA damage response with increased levels of RAD51 foci by treatment with acetaldehyde in hamster lung fibroblasts [49]. However, studies on the relationship between RAD51 and alcohol consumption in oral cancers require further investigation.

We observed that RAD51 protein was related to resistance to chemotherapy and radiotherapy We also observed that tumorsphere formation, a hallmark of cancer cell stemness, was increased in oral cancer cells overexpressing RAD51, while the expression of stemness markers, including CD44, CD133, Nanog, Oct4, and SOX2, was not changed by RAD51 expression. This suggested that RAD51-mediated treatment resistance in oral cancer occurs through mechanisms other than stem cell signaling and the role of RAD51 in the resistance to chemotherapy and radiotherapy requires further exploration, for example whole genome sequencing in the future.

RAD51 has been investigated as a therapeutic target in cancer treatment in recent years, and several small-molecule inhibitors of RAD51 have been reported [50]. B02 is a highly specific inhibitor of RAD51 that acts through direct binding [51, 52]. It impedes HR by disrupting RAD51 binding to DNA and inhibiting nucleoprotein filament formation [52]. B02 significantly enhances sensitivity of cancer cells to DNA-damaging agents, including the DNA cross-linking agents (cisplatin and mitomycin), topoisomerase 1 inhibitors (topotecan), and topoisomerase 2 inhibitors (doxorubicin) [53, 54]. As with previous studies on other cancer types, we discovered that the addition of B02 significantly increased the cytotoxicity of cisplatin in OECM1 oral cancer cells.

In addition, recent studies found that inhibition of CDK4/6 by Palbociclib when combined with irradiation increase treatment response in OSCC cells by inducing senescence, inhibiting DNA damage repair and reducing Rad51 and Ku80 expression [24]. This result consolidates the evidence that RAD51 is an important target to increase treatment efficacy when combined with other treatments. Moreover, an ongoing human phase I/II trial conducted in patients with advanced solid and hematologic cancers by using CYT-0851, a first-in-class small molecular inhibitor of RAD51-mediated DNA repair, showed that two of 10 evaluable patients had partial responses and additional two more patients experienced stable disease [55]. The encouraging result suggested that RAD51 could be a promising therapeutic target and further studies were warranted.

This study has some limitations. First, our study only included OSCC patients who received surgery as first line treatment and excluded metastatic OSCC patients. Therefore, the result could not represent all stages of OSCC patients. Second, we did not perform the positive control for IHC staining of RAD51 in this study; instead, we used an image as a positive reference from a previous study of esophageal cancer [56]. Third, the IHC scoring was determined by visual scoring from two investigators rather than automated IHC measurement. Therefore, the interpretation bias of IHC scoring from different investigators cannot be ruled out. Fourth, only clinical and in vitro studies were included in our study design and we did not perform in vivo animal study. Previous study reported that RAD51 inhibitor, RI-1, suppressed the growth of cervical cancer xenografts in vivo [57]. B02, another RAD51 inhibitor, in combination with cisplatin significantly inhibited MDA-MB-231 breast tumor growth in mouse xenografts [54]. Both studies showed that inhibition of RAD51 markedly suppressed tumor growth in mouse xenografts. Future studies are required to validate the in vivo activity of RAD51 on OSCC. Lastly, we didn’t clarify the detail mechanisms of RAD51-mediated treatment resistance in OSCC. In RAD51 overexpressed cells, the increased tumorsphere formation but without involvement of stemness markers suggested that other stem cell signaling pathway should be explored in the future.

## Conclusions

This study showed that RAD51 protein was a poor prognostic factor for patients with OSCC, especially those received adjuvant chemotherapy and radiotherapy. High RAD51 protein expression is correlated with resistance to chemotherapy and radiotherapy. A small-molecule inhibitor of RAD51, B02, significantly sensitized oral cancer cells to cisplatin treatment. Larger-scale studies are warranted to confirm whether combination therapy of chemotherapy/radiotherapy and drugs targeting RAD51 is applicable to solve treatment resistance issues and improve survival in OSCC patients.

### Electronic supplementary material

Below is the link to the electronic supplementary material.

Supplementary Material 1

Supplementary Material 2

Supplementary Material 3

Supplementary Material 4

Supplementary Material 5

Supplementary Material 6

## Data Availability

The datasets used and/or analyzed during the current study are available from the corresponding author on reasonable request.

## Abbreviations

OSCC: Oral squamous cell carcinoma
IARC: International Agency for Research on Cancer
GLOBOCAN: Global Cancer Observatory
DDR: DNA damage response
DSBs: Double-strand breaks
HR: Homologous recombination
HRR: Homologous recombination repair
NHEJ: Non-homologous end-joining
CDK: Cyclin-dependent kinases
AJCC: American Joint Cancer Committee on Cancer
FFPE: Formalin-fixed, paraffin-embedded
IHC: Immunohistochemistry
PCI: Positive stained-cell index
FBS: Fetal bovine serum
OD: Optical density
PBS: Phosphate-buffered saline
SD: Standard deviation
OS: Overall survival
PFS: Progression-free survival
HR: Hazard ratio
SCLC: Small cell lung cancer
