# Supplementary material for: RAD51 is a poor prognostic marker and a potential therapeutic target for oral squamous cell carcinoma
Source: Cancer Cell Int. 2023 Oct 5;23:231. doi: 10.1186/s12935-023-03071-w (PMC10552296; doi:10.1186/s12935-023-03071-w)
Supplement: Supplementary file 1 — Supplementary Material 1 [file 12935_2023_3071_MOESM1_ESM.docx]

**Table S.1 Baseline characteristics of 105 OSCC patients**

| **Characteristics** | **Results** |
| --- | --- |
| Male, N(%) | 97(92.4) |
| Age (years) (median, range) | 53(32-80) |
| Alcohol consumption, N(%) |  |
| Yes | 79(75.2) |
| No | 23(21.9) |
| Missing data | 3(2.9) |
| Betel nut chewing, N(%) |  |
| Yes | 85(81.0) |
| No | 17(16.2) |
| Missing data | 3(2.8) |
| Cigarette Smoking, N(%) |  |
| Yes | 82(78.1) |
| No | 20(19.0) |
| Missing data | 3(2.9) |
| T stage, N(%) |  |
| T1,2 | 79(75.3) |
| T3,4 | 26(24.7) |
| Lymph node metastasis, N(%) |  |
| Yes | 23(21.9) |
| No | 82(78.1) |
| Stage, N(%) |  |
| I | 41(39.0) |
| II | 22(21.0) |
| III | 12(11.4) |
| IV | 30(28.6) |
| Chemotherapy, N(%) |  |
| Yes | 43(41.0) |
| No | 62(59.0) |
| Radiotherapy, N(%) |  |
| Yes | 45(42.9) |
| No | 60(57.1) |
| RAD51 expression, N(%) |  |
| Low expression | 34 (32.4) |
| High expression | 71 (67.6) |

OSCC: oral squamous cell carcinoma

**Table S.2 The correlation between RAD51 expression and clinicopathological parameters**

| **Parameters** | **Low RAD51 expression** | | | **High RAD51expression** | | |
| --- | --- | --- | --- | --- | --- | --- |
|  |  | N | % | N | % | P value |
| Alcohol consumption | no | 12 | 36.4 | 11 | 15.9 |  |
|  | yes | 21 | 63.6 | 58 | 84.1 | 0.021* |
| Betel nut chewing | no | 7 | 21.2 | 10 | 14.5 |  |
|  | yes | 26 | 78.8 | 59 | 85.5 | 0.394 |
| Cigarette smoking | no | 10 | 30.3 | 10 | 14.5 |  |
|  | yes | 23 | 69.7 | 59 | 85.5 | 0.060 |
| Sex | Male | 29 | 85.3 | 68 | 95.8 |  |
|  | Female | 5 | 14.7 | 3 | 4.2 | 0.109 |
| Age | ≤50 | 16 | 47.1 | 27 | 38.0 |  |
|  | >50 | 18 | 52.9 | 44 | 62.0 | 0.379 |
| T stage | T1 | 16 | 47.1 | 34 | 47.9 |  |
|  | T2 | 9 | 26.5 | 20 | 28.2 |  |
|  | T3 | 3 | 8.8 | 3 | 4.2 |  |
|  | T4 | 6 | 17.6 | 14 | 19.7 | 0.818 |
| N stage | LN (-) | 30 | 88.2 | 52 | 73.2 |  |
|  | LN(+) | 4 | 11.8 | 19 | 26.8 | 0.129 |
| Stage | I, II, | 22 | 64.7 | 41 | 57.5 |  |
|  | III, IV | 12 | 35.3 | 30 | 42.3 | 0.530 |
| Tumor grade | G1 | 27 | 84.4 | 60 | 88.2 |  |
|  | G2 | 5 | 15.6 | 8 | 11.8 | 0.592 |

*T: tumor, N: lymph node, LN: lymph node
