# Supplementary figures and images for: RAD51 is a poor prognostic marker and a potential therapeutic target for oral squamous cell carcinoma
Source: Cancer Cell Int. 2023 Oct 5;23:231. doi: 10.1186/s12935-023-03071-w (PMC10552296; doi:10.1186/s12935-023-03071-w)

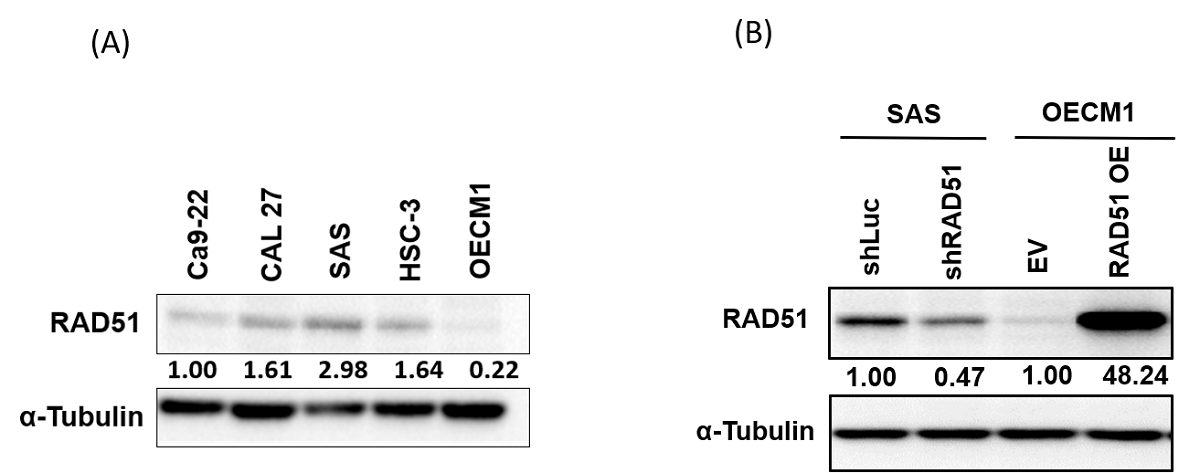

Supplement: Supplementary file 2 — Supplementary Material 2 [file 12935_2023_3071_MOESM2_ESM.png]

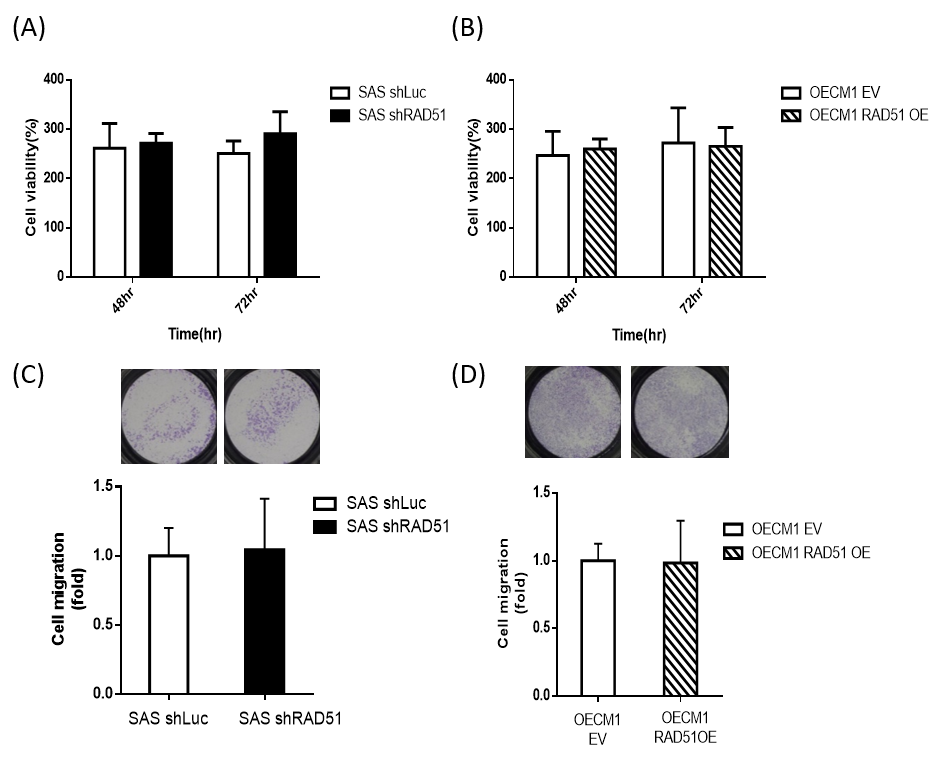

Supplement: Supplementary file 3 — Supplementary Material 3 [file 12935_2023_3071_MOESM3_ESM.png]

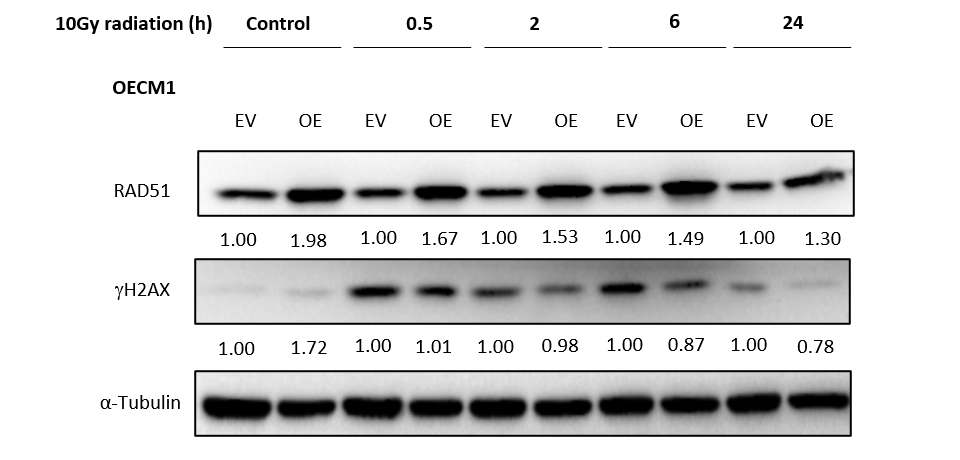

Supplement: Supplementary file 4 — Supplementary Material 4 [file 12935_2023_3071_MOESM4_ESM.png]

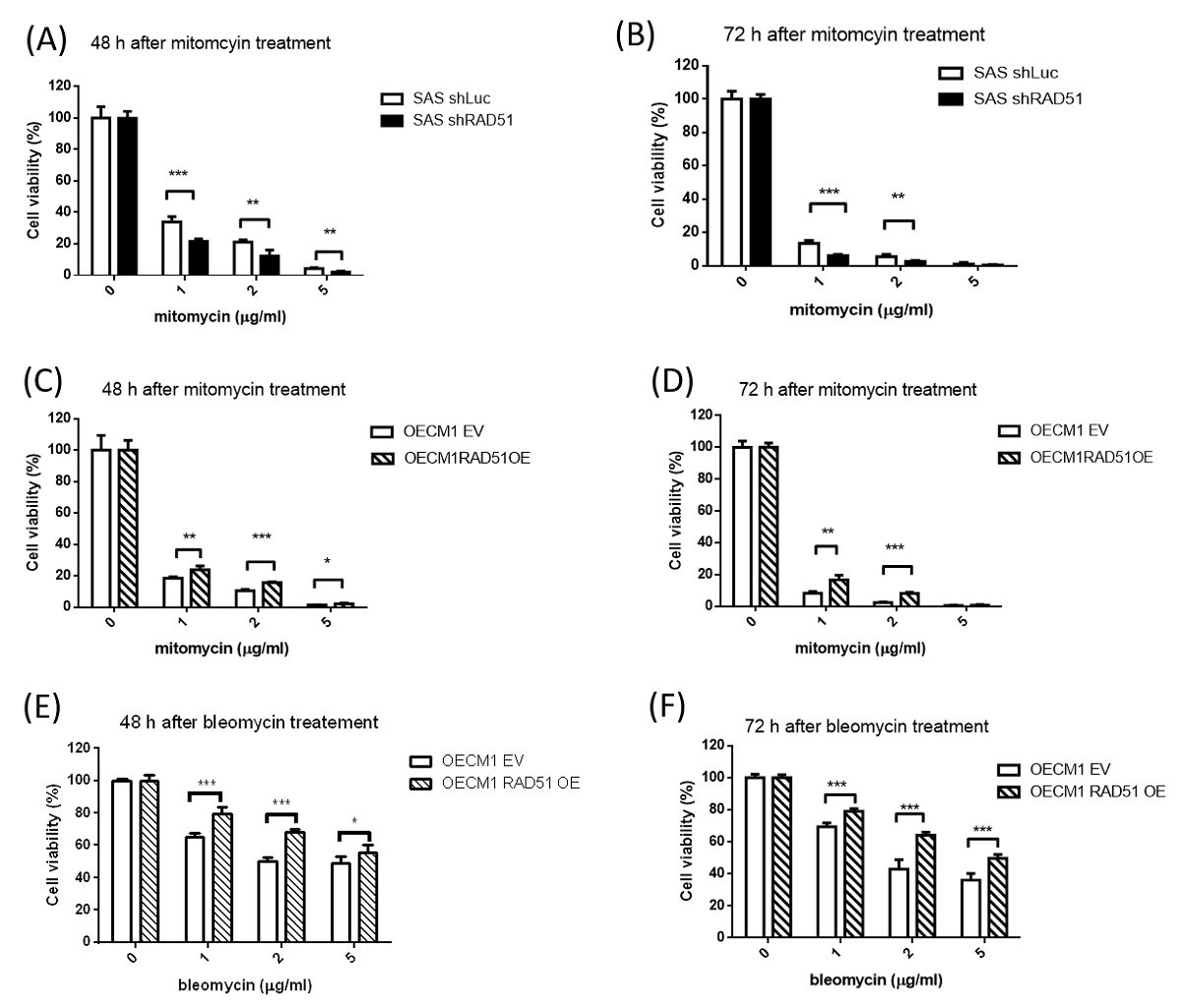

Supplement: Supplementary file 5 — Supplementary Material 5 [file 12935_2023_3071_MOESM5_ESM.png]

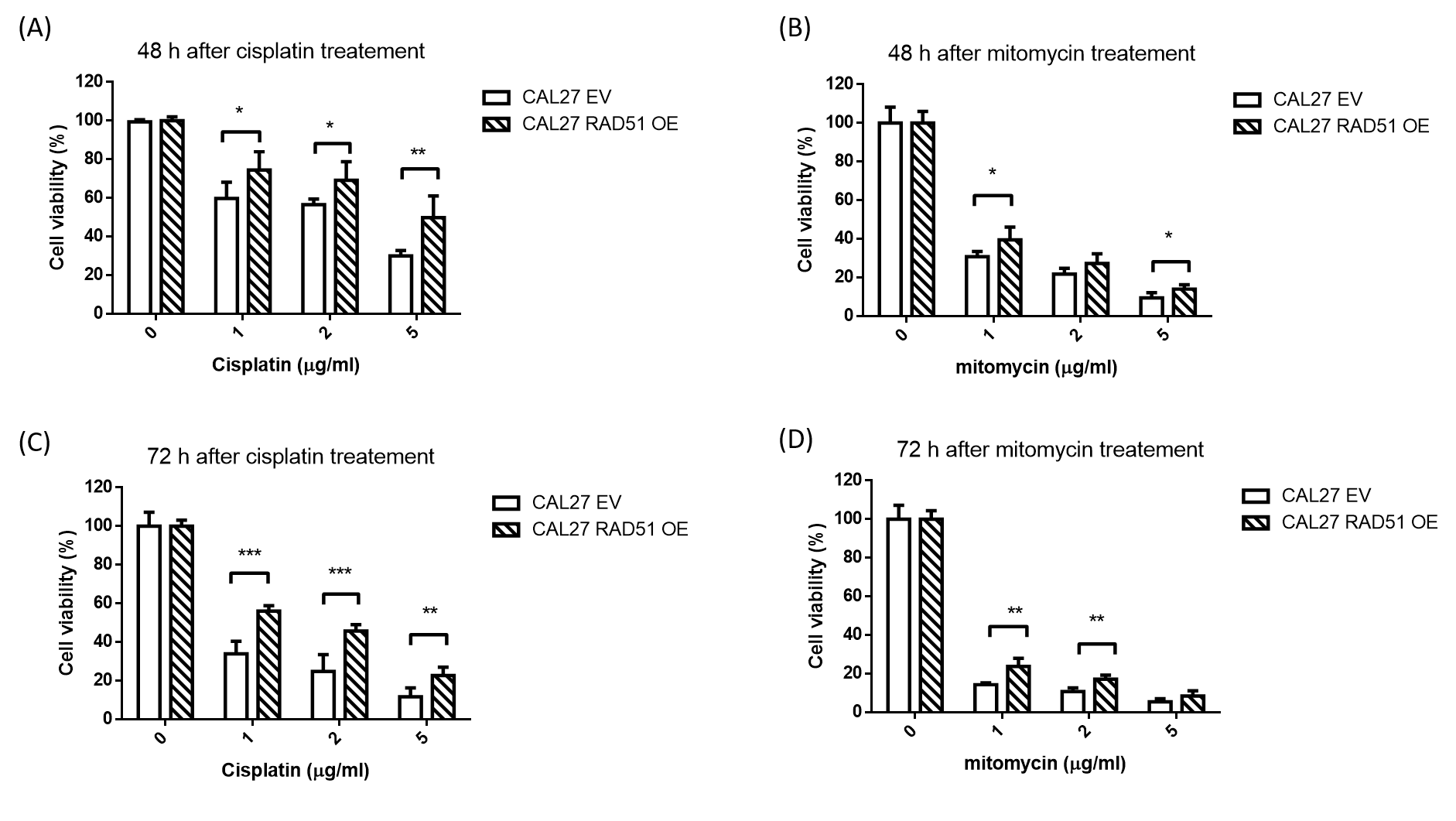

Supplement: Supplementary file 6 — Supplementary Material 6 [file 12935_2023_3071_MOESM6_ESM.png]
